# Genome sequence of the filamentous, gliding *Thiothrix nivea* neotype strain (JP2^T^)

**DOI:** 10.4056/sigs.2344929

**Published:** 2011-12-30

**Authors:** Alla Lapidus, Matt Nolan, Susan Lucas, Tijana Glavina Del Rio, Hope Tice, Jan-Fang Cheng, Roxanne Tapia, Cliff Han, Lynne Goodwin, Sam Pitluck, Konstantinos Liolios, Ioanna Pagani, Natalia Ivanova, Marcel Huntemann, Konstantinos Mavromatis, Natalia Mikhailova, Amrita Pati, Amy Chen, Krishna Palaniappan, Miriam Land, Evelyne-Marie Brambilla, Manfred Rohde, Birte Abt, Susanne Verbarg, Markus Göker, James Bristow, Jonathan A. Eisen, Victor Markowitz, Philip Hugenholtz, Nikos C. Kyrpides, Hans-Peter Klenk, Tanja Woyke

**Affiliations:** 1 DOE Joint Genome Institute, Walnut Creek, California, USA; 2 Los Alamos National Laboratory, Bioscience Division, Los Alamos, New Mexico, USA; 3 Biological Data Management and Technology Center, Lawrence Berkeley National Laboratory, Berkeley, California, USA; 4 Oak Ridge National Laboratory, Oak Ridge, Tennessee, USA; 5 Leibniz Institute DSMZ - German Collection of Microorganisms and Cell Cultures, Braunschweig, Germany; 6 HZI – Helmholtz Centre for Infection Research, Braunschweig, Germany; 7 University of California Davis Genome Center, Davis, California, USA; 8 Australian Centre for Ecogenomics, School of Chemistry and Molecular Biosciences, The University of Queensland, Brisbane, Australia

**Keywords:** strictly aerobic, gliding motility, Gram-negative, mesophile, sheath, filaments, sulfur granules, *Thiotrichaceae*, GEBA

## Abstract

*Thiothrix nivea* (Rabenhorst 1865) Winogradsky 1888 (Approved Lists 1980) emend. Larkin and Shinabarger 1983 is the type species of the genus *Thiothrix* in the family *Thiotrichaceae*. The species is of interest not only because of its isolated location in the yet to be genomically characterized region of the tree of life, but also because of its life-style with gliding gonidia, the multilayer sheath, rosettes, and the embedded sulfur granules. Strain JP2^T^ is the neotype strain of the species which was first observed by Rabenhorst in 1865 and later reclassified by Winogradsky in 1888 into the then novel genus *Thiothrix*. This is the first completed (improved-high-quality-draft) genome sequence to be published of a member of the family *Thiotrichaceae*. The genome in its current assembly consists of 15 contigs in four scaffolds with a total of 4,691,711 bp bearing 4,542 protein-coding and 52 RNA genes and is a part of the *** G****enomic* ***E****ncyclopedia of* ***B****acteria and* ***A****rchaea * project.

## Introduction

Strain JP2^T^ (= DSM 5205 = ATCC 35100) is the type strain of *Thiothrix nivea* [1,2] which is the type species of the genus *Thiothrix* [1,2]. Cultures of the species were first observed and classified as “*Beggiatoa nivea*” in 1865 by Rabenhorst [3] and later (1888) placed into the novel genus *Thiothrix* by Winogradsky [2]. The species was included on the Approved List of Bacterial Names Amended edition in 1980 [4]. Axenic cultures isolated from sulfide-containing well water became available in 1980 through the work of J. M. Larkin [5], with the formal description of strain JP2^T^ as the neotype strain of the species *T. nivea* in 1983 [1], as well as strain JP1 as a reference strain within the species [1]. The generic name derives from the Neo-Greek words *theion*, sulfur, and *thrix*, hair [6]. The species epithet is derived from the Latin word *nivea* snow-white [6]. The species became well known for its sulfur granules, the gliding motility and the typical rosettes [1], which were first observed by Winogradsky [2]. Here we present a summary classification and a set of features for *T. nivea* JP2^T^, together with the description of the complete genomic sequencing and annotation.

## Classification and features

A representative genomic 16S rRNA sequence of *T. nivea* JP2^T^ was compared using NCBI BLAST [7] under default settings (e.g., considering only the high-scoring segment pairs (HSPs) from the best 250 hits) with the most recent release of the Greengenes database [8] and the relative frequencies of taxa and keywords (reduced to their stem [9]) were determined, weighted by BLAST scores. The most frequently occurring genus was *Thiothrix* (100.0%, 12 hits in total). Regarding the single hit to sequences from members of the species, the average identity within HSPs was 99.5%, whereas the average coverage by HSPs was 99.4%. Regarding the four hits to sequences from other members of the genus, the average identity within HSPs was 94.2%, whereas the average coverage by HSPs was 96.0%. Among all other species, the one yielding the highest score was, *Thiothrix fructosivorans* (GU269554) which corresponded to an identity of 94.5% and an HSP coverage of 100.0%. (Note that the Greengenes database uses the INSDC (= EMBL/NCBI/DDBJ) annotation, which is not an authoritative source for nomenclature or classification.) The highest-scoring environmental sequence was AM490765 ('Linking and functional nutrient spiraling mats (USA) microbial mat sulfidic cave spring Lower Kane Cave Big Horn LKC22 clone SS LKC22 UB32'), which showed an identity of 96.7% and an HSP coverage of 100.0%. The most frequently occurring keywords within the labels of environmental samples which yielded hits were 'sulfid' (4.2%), 'microbi' (4.0%), 'biofilm' (3.4%), 'cave' (2.8%) and 'karst' (2.7%) (238 hits in total). Environmental samples which yielded hits of a higher score than the highest scoring species were not found. These keywords reflect the ecological properties reported for the species and strain JP2^T^ in the original description [1,2].

**Figure 1:**
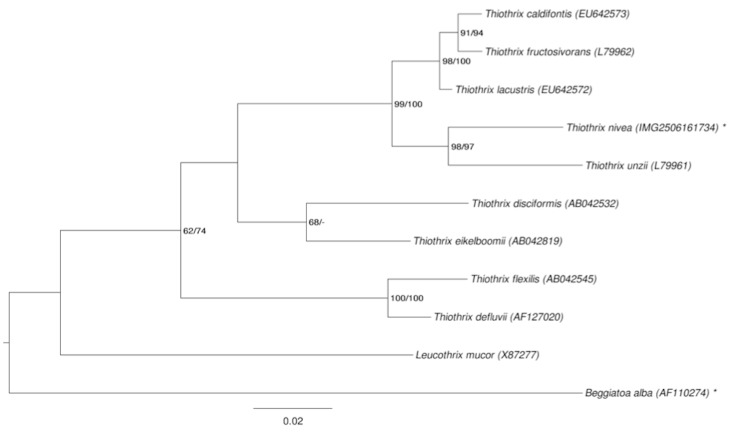
Phylogenetic tree highlighting the position of *T. nivea* relative to the other type strains within the family *Thiotrichaceae*. The tree was inferred from 1,332 aligned characters [10,11] of the 16S rRNA gene sequence under the maximum likelihood (ML) criterion [12]. Rooting was done initially using the midpoint method [13] and then checked for its agreement with the current classification (Table 1). The branches are scaled in terms of the expected number of substitutions per site. Numbers adjacent to the branches are support values from 200 ML bootstrap replicates [14] (left) and from 1,000 maximum parsimony bootstrap replicates [15] (right) if larger than 60%. Lineages with type strain genome sequencing projects registered in GOLD [16] are labeled with one asterisk, those also listed as 'Complete and Published' with two asterisks.

Figure 1 shows the phylogenetic neighborhood of *T. nivea* in a 16S rRNA based tree. The sequences of the two identical 16S rRNA gene copies in the genome do not differ from the previously published 16S rRNA sequence (L40993), which contains six ambiguous base calls.

**Table 1:** Classification and general features of *T. nivea* JP2^T^ according to the MIGS recommendations [17] and the NamesforLife database [18].

MIGS ID	Property	Term	Evidence code
	Current classification	Domain *Bacteria*	TAS [19]
		Phylum “*Proteobacteria*”	TAS [20,21]
		Class *Gammaproteobacteria*	TAS [22]
		Order *Thiotrichales*	TAS [22,23]
		Family *Thiotrichaceae*	TAS [22,24]
		Genus *Thiothrix*	TAS [2,4,25-27]
		Species *Thiothrix nivea*	TAS [1,5]
		Type strain JP2	TAS [1]
	Gram stain	negative	TAS [1]
	Cell shape	rod-shaped, filaments with a sheath, rosettes	TAS [1]
	Motility	gliding	TAS [1]
	Sporulation	not reported	
	Temperature range	mesophilic, 6-34°C	TAS [1]
	Optimum temperature	25-30°C	TAS [1]
	Salinity	not reported	
MIGS-22	Oxygen requirement	strictly aerobic	TAS [1]
	Carbon source	acetate, malate, pyruvate, oxalacetate	TAS [1]
	Energy metabolism	chemolithotroph	NAS
MIGS-6	Habitat	spring-generated, flowing, H_2_S-enriched waters, deep sea hydrothermal vents	TAS [28,29]
MIGS-15	Biotic relationship	free-living	TAS [1]
MIGS-14	Pathogenicity	none	TAS [1]
	Biosafety level	1	TAS [30]
	Isolation	H_2_S-enriched well water	TAS [1]
MIGS-4	Geographic location	John Pennycamp State Park, Key Largo, FL, USA	TAS [1]
MIGS-5	Sample collection time	1983 or before	NAS
MIGS-4.1	Latitude	25.13	NAS
MIGS-4.2	Longitude	-80.41	NAS
MIGS-4.3	Depth	not reported	
MIGS-4.4	Altitude	not reported	

Evidence codes - TAS: Traceable Author Statement (i.e., a direct report exists in the literature); NAS: Non-traceable Author Statement (i.e., not directly observed for the living, isolated sample, but based on a generally accepted property for the species, or anecdotal evidence). These evidence codes are from of the Gene Ontology project [31].

**Figure 2:**
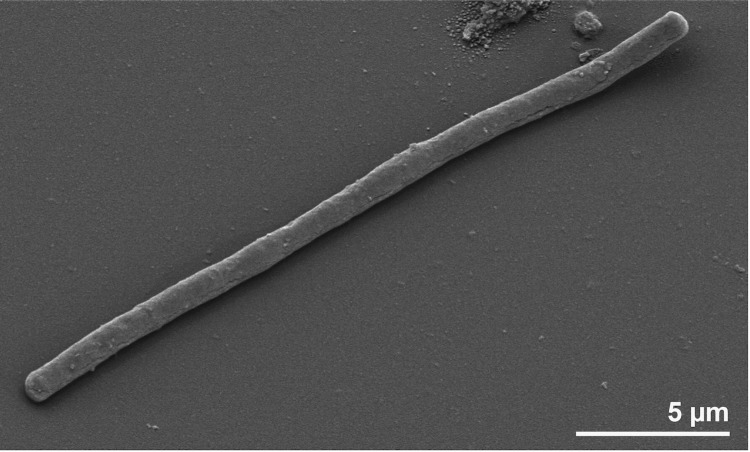
Scanning electron micrograph of *T. nivea* JP2^T^

Cells of strain JP2^T^ are rod shaped with various lengths (Figure 2). Cultures of *T. nivea* contain gliding gonidia, filaments and rosettes (= aggregations of gonidial cells, not visible in Figure 2) [1]. The presence of a sheath was first reported in the 19^th^ century [2] and later confirmed for the neotype strain [1]. The sheath contains several separate layers [1] of so far unknown structure. Motility was observed, but no flagella [1]. Numerous genes allocated to the functional role category motility were identified in the genome (see below). Many of these genes might be involved in the formation of the polar located fimbriae [32]. The typical rosettes generated by *T. nivea* are known from sulfide-containing waters [1,2]. Sulfur granules are invaginated by the cells, as reported in detail by Larkin and Shinabarger [1]. Strain JP2^T^ stains Gram-negative, and grows only aerobically, best within a temperature range of 20 – 30°C [1]. Both the neotype strain and reference strain JP1 produce oxidase, but not catalase. The strains also produce poly-β-hydroxybutyrate [1]. Strain JP2^T^ uses only four carbon sources; acetate, malate, pyruvate and oxalacetate [1]. Ammonia and nitrate (but not nitrite) are used as sole nitrogen sources [1]. The sole sulfur sources are sulfide and thiosulfate. What remains unresolved, based on the literature is whether or not *T. nivea* is autotrophic, obtaining carbon from CO_2_ and energy *via* oxidation of sulfide as reported by Winogradsky [13] or not, as reported by Larkin and Shinabarger [1]. In the case in which strain JP2^T^ could use CO_2_ as a carbon source as well as acetate, malate, pyruvate and oxalacetate, while oxidizing the reduced sulfur compounds, it could be considered to be a mixotroph [1].

### Chemotaxonomy

There are no chemotaxonomic data on cell wall structure, cellular lipids, quinones or polar lipids of strain JP2^T^.

## Genome sequencing and annotation

### Genome project history

This organism was selected for sequencing on the basis of its phylogenetic position [33], and is part of the *** G****enomic* ***E****ncyclopedia of* ***B****acteria and* ***A****rchaea * project [34]. The genome project is deposited in the Genome On Line Database [16] and the complete genome sequence is deposited in GenBank. Sequencing, finishing and annotation were performed by the DOE Joint Genome Institute (JGI). A summary of the project information is shown in Table 2.

**Table 2:** Genome sequencing project information

**MIGS ID**	**Property**	**Term**
MIGS-31	Finishing quality	Improved-high-quality-draft
MIGS-28	Libraries used	Three genomic libraries: one 454 pyrosequence standard library, one 454 PE library (12 kb insert size), one Illumina library
MIGS-29	Sequencing platforms	Illumina GAii, 454 GS FLX Titanium
MIGS-31.2	Sequencing coverage	111.5 × Illumina; 28.9 × pyrosequence
MIGS-30	Assemblers	Newbler version 2.3-PreRelease-6/30/2009, Velvet version 1.0.13, phrap SPS-4.24
MIGS-32	Gene calling method	Prodigal 1.4, GenePRIMP
	INSDC ID	Not yet available
	Genbank Date of Release	Not yet available
	GOLD ID	Gi03023
	NCBI project ID	51139
	Database: IMG-GEBA	2506520049
MIGS-13	Source material identifier	DSM 5205
	Project relevance	Tree of Life, GEBA

### Growth conditions and DNA isolation

*T. nivea* JP2^T^, DSM 5205, was grown in DSMZ medium 1300 (*Thiothrix* Medium) [35] at 25°C. DNA was isolated from 0.5-1 g of cell paste using Jetflex Genomic DNA Purification Kit (GENOMED 600100) following the standard protocol as recommended by the manufacturer, but adding 10µl proteinase K for one hour extended lysis at 58°C. DNA is available through the DNA Bank Network [36].

### Genome sequencing and assembly

The genome was sequenced using a combination of Illumina and 454 sequencing platforms. All general aspects of library construction and sequencing can be found at the JGI website [37]. Pyrosequencing reads were assembled using the Newbler assembler (Roche). The initial Newbler assembly consisting of 269 contigs in four scaffolds was converted into a phrap assembly by [38] making fake reads from the consensus to collect the read pairs in the 454 paired end library. Illumina GAii sequencing data (518.8 Mb) was assembled with Velvet [39], and the consensus sequences were shredded into 1.5 kb overlapped fake reads and assembled together with the 454 data. The 454 draft assembly was based on 162.5 Mb 454 draft data and all of the 454 paired end data. Newbler parameters are -consed -a 50 -l 350 -g -m -ml 20. The Phred/Phrap/Consed software package [38] was used for sequence assembly and quality assessment in the subsequent finishing process. After the shotgun stage, reads were assembled with parallel phrap (High Performance Software, LLC). Possible mis-assemblies were corrected with gapResolution [37], Dupfinisher, or sequencing cloned bridging PCR fragments with subcloning or transposon bombing (Epicentre Biotechnologies, Madison, WI). Gaps between contigs were closed by editing in Consed, by PCR and by Bubble PCR primer walks (J.-F. Chang, unpublished). A total of 632 additional reactions were necessary to close gaps and to raise the quality of the final sequence. Illumina reads were also used to correct potential base errors and increase consensus quality using a software Polisher developed at JGI [40]. This genome is not finished. The improved high quality draft consists of 15 contigs in four scaffolds. Some mis-assemblies are possible in the final assembly. Together, the combination of the Illumina and 454 sequencing platforms provided 140.4 × coverage of the genome. The final assembly contained 444,417 pyrosequence and 14,381,947 Illumina reads.

### Genome annotation

Genes were identified using Prodigal [41] as part of the Oak Ridge National Laboratory genome annotation pipeline, followed by a round of manual curation using the JGI GenePRIMP pipeline [42]. The predicted CDSs were translated and used to search the National Center for Biotechnology Information (NCBI) non-redundant database, UniProt, TIGR-Fam, Pfam, PRIAM, KEGG, COG, and InterPro databases. Additional gene prediction analysis and functional annotation was performed within the Integrated Microbial Genomes - Expert Review (IMG-ER) platform [43].

## Genome properties

The genome consists in the current assembly of 15 contigs in four scaffolds with a length of 5,599 bp, 7,015 bp, 40,927 bp, and 4,638,170 bp, respectively, and a G+C content of 54.9% (Table 3). Of the 4,594 genes predicted, 4,542 were protein-coding genes, and 52 RNAs; 213 pseudogenes were also identified. The majority of the protein-coding genes (98.8%) were annotated as hypothetical proteins. The distribution of genes into COGs functional categories is presented in Table 4.

**Table 3:** Genome Statistics

**Attribute**	**Value**	**% of Total**
Genome size (bp)	4,691,711	100.00%
DNA coding region (bp)	4,147,061	88.39%
DNA G+C content (bp)	2,573,778	54.87%
Number of scaffolds	4	
Number of contigs	15	
Total genes	4,594	100.00%
RNA genes	52	1.15%
rRNA operons	2	
Protein-coding genes	4,542	98.85%
Pseudo genes	213	4.64%
Genes with function prediction	2,918	63.52%
Genes in paralog clusters	2,282	49.67%
Genes assigned to COGs	3,275	71.29%
Genes assigned Pfam domains	3,338	72.66%
Genes with signal peptides	971	21.14%
Genes with transmembrane helices	1,027	22.36%
CRISPR repeats	4	

**Table 4:** Number of genes associated with the general COG functional categories

**Code**	**value**	**%age**	**Description**
J	168	4.7	Translation, ribosomal structure and biogenesis
A	3	0.1	RNA processing and modification
K	211	5.9	Transcription
L	276	7.7	Replication, recombination and repair
B	1	0.0	Chromatin structure and dynamics
D	48	1.3	Cell cycle control, cell division, chromosome partitioning
Y	0	0.0	Nuclear structure
V	70	2.0	Defense mechanisms
T	208	5.8	Signal transduction mechanisms
M	266	7.4	Cell wall/membrane/envelope biogenesis
N	56	1.6	Cell motility
Z	0	0.0	Cytoskeleton
W	0	0.0	Extracellular structures
U	102	2.9	Intracellular trafficking, secretion, and vesicular transport
O	162	4.5	Posttranslational modification, protein turnover, chaperones
C	289	8.1	Energy production and conversion
G	127	3.6	Carbohydrate transport and metabolism
E	210	5.9	Amino acid transport and metabolism
F	59	1.7	Nucleotide transport and metabolism
H	143	4.0	Coenzyme transport and metabolism
I	82	2.3	Lipid transport and metabolism
P	196	5.5	Inorganic ion transport and metabolism
Q	58	1.6	Secondary metabolites biosynthesis, transport and catabolism
R	407	11.4	General function prediction only
S	437	12.2	Function unknown
-	1,319	28.7	Not in COGs

## Insight into the genome sequence

The genomic basis for gliding motility is not yet completely resolved, but the requirement of the genes *gldA*, *gldF* and *gldG* was described [44]. Genes *gldA*, *gldF* and *gldG* exhibit a high degree of sequence similarity to components of ABC transporters [44]. A closer examination of the JP2^T^ genome revealed a region of three genes (Thini_0004.00016790, Thini_0004.00016780, Thini_0004.00016770, currently annotated as ABC-type uncharacterized transport system), for which the derived protein sequences show high similarity to GldA, GldF and GldG of *Bdellovibrio bacteriovorus* HD100 (Bd1023, Bd1024 and Bd1025) [45]. The requirement of *gldA* for gliding motility was experimentally shown for *Flavobacterium johnsoniae*. A non-motile mutant lacking the intact *gldA* gene was complemented by a vector carrying an intact *gldA* gene. The motility of the mutant was restored [46].

While we were able to locate a phosphoenolpyruvate carboxylase gene in the genome, (Thini_0004.00035050), we could not identify a gene for malate dehydrogenase. Unless *T. nivea* encodes a malate dehydrogenase that is not homologous to other malate dehydrogenases, we can not confirm for the neotype strain the genomic basis for the speculation that the original *T. nivea* culture can fix CO_2_ as reported by Winogradsky [13] and is a mixotroph [1].
